# Delta variant of COVID-19: A simple explanation

**DOI:** 10.5339/qmj.2021.49

**Published:** 2021-10-05

**Authors:** Mohamed A. Hendaus, Fatima A. Jomha

**Affiliations:** ^1^Department of Pediatrics, Sidra Medicine, Qatar E-mail: mhendaus@yahoo.com; ^2^Weill Cornell Medicine, Qatar; ^3^Department of Pharmacy, Sidra Medicine, Qatar

## Abstract

Severe acute respiratory syndrome coronavirus 2, the virus that causes coronavirus disease (COVID-19), has undergone numerous mutations since its initial identification, leading to challenges in controlling the pandemic. Till date, several variants of concern have been identified. However, currently, the Delta variant (B.1.617.2) is the most dreaded one owing to its enhanced transmissibility and increased virulence. In addition, this variant can potentially facilitate fusion of the spike protein to cells or inhibit antibodies from binding to it. In this commentary, we have simplified the complexity of the nomenclature of variants related to COVID-19, concentrating on the Delta variant including its transmissibility, response to vaccines, and prevention.

## Introduction

Coronavirus disease (COVID-19) has had a detrimental outcome on the global population and has caused millions of deaths worldwide. In addition, COVID-19 appears to the leading world health crisis since the influenza pandemic of 1918.^1^

Severe acute respiratory syndrome coronavirus 2 (SARS-CoV-2), the virus that causes COVID-19, has undergone numerous mutations since its initial identification, leading to challenges in controlling the pandemic.^2^

So far, several variants have been of concern. The Alpha(B.1.1.7) variant was identified in United Kingdom in late December 2020, Beta(B.1.351) variant in South Africa in December 2020, and Gamma (P.1) variant in Brazil in early January 2021. The Delta(B.1.617.2) variant was first reported in India in December 2020 and is currently the most dreaded variant owing to its enhanced transmissibility and increased virulence.^3^

In this commentary, we simplify the complex nomenclature of COVID-19-causing viral variants. We have also focused on the Delta variant including its transmissibility, response to vaccines, and prevention.

## Reasons for mutations

Viruses, especially those with ribonucleic acid as their genetic material, frequently mutate, including SARS-CoV-2 and influenza virus. When a virus is circulating extensively and is causing an illness, the probability of the virus mutating augments. The more chances a virus has to expand, the more it replicates, and more are its chances to undergo changes. An error in this copying process prompts a mutation. Most viral mutations have minimal to no impact on the virus's capability to worsen illness. However, depending on where the mutations are located in the virus's genetic material, they may affect the virus's properties, such as virulence, immune escape, or transmission.^4^

It is estimated that SARS-CoV-2 can make more than 1 billion copies (10^9^–10^11^) of itself after infecting an individual. During this rapid replication, some mistakes occur; these are manifested as mutations inside the genetic material of the virus.^5^

## Spike protein mutations

**Figure 1.**
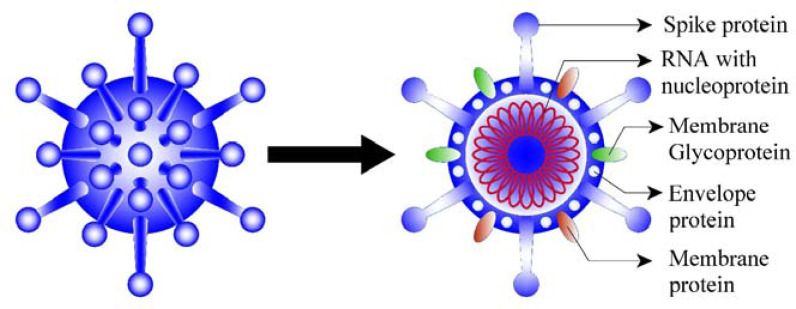
Structure of SARS-CoV-2

SARS-CoV-2 is covered with fatty membrane proteins (or glycoproteins) that can allow the virus to fuse to the host's cell membrane. The spike protein is located on SARS-CoV-2 shell and is made of a linear chain of approximately 1,300 amino acids. This protein relates to the host cells, such as the pulmonary and parabronchial epithelial cells,^6^ and helps the virus enter through the epithelial cell membrane (Figure 1).

SARS-CoV-2 eventually binds with angiotensin-converting enzyme 2, which plays a crucial role in the commencement of COVID-19 infection.^7,8,9^

Mutations, which instigate substantial changes in the spike protein, can be of concern because they can induce changes in the biochemical characteristics and structure of the virus. These changes facilitate the spike protein to fuse to cells or inhibit antibodies from binding to it.^10^

Selective sweeps in the spike protein, have likely played a crucial role in the adaptive evolution of SARS-CoV-2.^11^ Kang et al^11^ showed that a single spike protein mutation might be responsible for the transfer of this coronavirus from animals to humans. This study also showed that a single mutation (alanine replacing threonine) was sufficient to allow transmission from bats to humans. Moreover, this replacement led to a simple mutation (T372A) resulting in an easier access of the virus into the human host cell.

## Classifications of COVID-19 mutations

In 2020, the World Health Organization (WHO) characterized SARS-CoV-2 variants into variants of interest (VOIs) and variants of concern (VOCs). This classification was aimed at prioritizing worldwide monitoring and research and eventually managing appropriate response to the COVID-19 pandemic. WHO defines a VOI as a SARS-CoV-2 that has phenotypically and genetically changed and can affect the disease relentlessness and community transmission.^12^

The current identified VIOs are as follows: Iota (B.1.526), Eta (B.1.525), Kappa (B.1.617.1), and Lambda (C.37).^13^

The currently identified VOCs include the Alpha (B.1.1.7), Beta (B.1.351), Gamma (P.1), and Delta (B.1.617.2).^3^

VOIs can be VOCs if there is a rise in the virus transmissibility; augmentation of fatalities; and reduction in the efficiency of vaccines, treatments, and other health measures.^12^

WHO has recommended using the Greek alphabet for VOCs and VOIs to ensure that the labels being used are “easy to pronounce” and “non-stigmatizing” to the countries where they were first identified or are thought to be originating from.^2^

## Nomenclature of lineages

There is no collective method to sub-classify viruses below the level of a virus species. Usually, genetic diversity can be sub-classified into distinct clades. These clades may be referred to by a variety of terms, such as “genotypes” or “subtypes” 1to reflect an effort to divide pathogen phylogeny and genetic diversity into their divergent types.^14^

The major lineage labels of SARS-CoV-2 begin with a letter, and the current lineages represented are A and B.^14^ This system of categorization is proposed only for tracking currently circulating lineages. To demonstrate phylogenetic evidence in a lineage, Rambault et al^15^ proposed the following criteria:

(1) A new lineage displays one or more mutual nucleotide differences from the ancestral lineage.
(2) A new lineage includes at least five genomes with >95% of the genome sequenced.
(3) Genomes within the lineage have a minimum of one shared nucleotide change among them.
(4) A resampling analysis (bootstrap) value >70% for the lineage-defining node.

The most common lineages appearing in the literature start with the letter B, as lineage B has been sequenced and published first (end of 2019). When the sequence of lineage gets too lengthy, the digit “l” is added as an alias to shorten the lineage name. For instance, the VOI spreading in Brazil is known as P.1 rather than B.1.1.28.1.

## The Delta variant (B.1.617.2):

The B.1.617.2 (Delta) variant was initially identified in India in December 2020 and eventually in numerous countries across the six continents. Nowadays, several mutations in the Delta variant spike protein (L452R, tbl478K, D614G, tbl19R, Δ157-158, P681R, and D950N) have been identified.^16^

Several of these mutations may affect immune responses, especially at the level of antigenic regions of receptor-binding proteins (452 and 478), permitting better attachment to the receptor cells and evading immunity more easily.^17^

As indicated on the Centers for Disease Control and Prevention website, the Delta variant has increased transmissibility, with potentially decreased neutralization by sera after vaccination, and with possibly decreased neutralization by some emergency use authorization (EUA) monoclonal antibody treatments.^18^

## R naught value and high infectiveness of Delta variant

Recently, Li et al^19^ studied *the transmissibility of the Delta SARS-CoV-2 variant* (B.1.617.2) in mainland China. The study identified 167 patients with Delta variant infections, with all of them being traced back to the index case. Data were retrieved from daily, sequential polymerase chain reaction testing of the isolated subjects. The authors showed that the viral load of the first positive test of patients with Delta variant infections was ∼1000 times higher than that of the patients with 19A/19B strain infection, which caused the epidemic in early 2020. This insinuates the impending faster viral replication rate and more infectiousness of the Delta variant (B.1.617.2) in the initial phase of the infection.

R_0_ (R naught) is an epidemiological term indicating how contagious the infectious disease is. It is a reproduction number to measure the transmissibility of infectious agents. R_0_ is derived from the period of infectivity after infection in the patient, the mode of transmission, and the contact rate.^20^

Reportedly, R_0_ of the original COVID-19 strain found in Wuhan was 2.4–2.6. Ensuing data has shown that the R_0_ for the Alpha strain was 4–5 and for the Delta (B.1.617.2) strain was 5–8. This implies that a person infected with the Delta variant of SARS-CoV-2 can transmit the infection to eight people. It also indicates that the Delta variant could be twice (even more) transmissible as the original COVID-19 strain.^21^

These data signify that the Delta variant of SARS-CoV-2 (B.1.617.2) is more infectious than smallpox, which has an R_0_ of 3.5–4.6.^22^ Hence, it is crucial for people to comply with physical distancing and other precautionary measures to better take control of the infection caused by the Delta variant_._

## Delta variant (B.1.617.2) and reduced vaccine efficiency

Vaccine efficacy showed modest differences against the Delta variant and the Alpha variant after the administration of two doses.^23^ However, these differences in vaccine effectiveness were more prominent after the administration of the first dose. A recent study published in the New England Journal of Medicine included a total of 19,109 patients (Alpha variant  = 14,837 and Delta variant  = 4272). It demonstrated that the efficacy after one dose of Oxford/AstraZeneca COVID-19 vaccine (ChAdOx1) or Pfizer–BioNTech COVID-19 vaccine (BNT162b2) was particularly lower in individuals infected with the Delta variant (30.7%; 95% confidence interval [CI]: 25.2–35.7) than in those infected with the Alpha variant (48.7%; 95% CI: 45.5–51.7).

After administering two vaccine doses, the effectiveness of the BNT162b2 vaccine was 93.7% (95% CI: 91.6–95.3) among patients infected with the Alpha variant compared with 88.0% (95% CI: 85.3–90.1) among those infected with the Delta variant (B.1.617.2).

The same study showed that for the ChAdOx1 nCoV-19 vaccine, the efficiency of two doses was 74.5% (95% CI: 68.4–79.4) in individuals infected with the Alpha variant and 67.0% (95% CI: 61.3–71.8) in those infected with the Delta variant (B.1.617.2).^23^

## Preventing new variants from emergence

Halting the spread at the source remains the key. Regularly used precautions employed since the beginning of the pandemic should be strictly followed. These measures include physical distancing, avoiding closed places or crowds, wearing a face mask or face shield, and cleaning hands frequently. By reducing the amount of viral transmissibility, the odds of a new variant emerging are low. Making the COVID-19 vaccine available globally is crucial in ensuring herd immunity.^24^

## Conclusion

The Delta variant (B.1.617.2) is characterized by its increased transmissibility, potential reduced neutralization by sera after vaccination, and possibly decreased neutralization by some EUA monoclonal antibody treatments. By reducing the amount of viral transmissibility, the odds of a new variant emerging are low. Making the COVID-19 vaccine available globally is crucial in ensuring herd immunity.

### Acknowledgment

Special appreciation for architect Riad Younes for developing the figure.

### Disclosure statement

The authors have no potential conflicts of interest relevant to this article to disclose.
